# Functional Heterologous Protein Expression by Genetically Engineered Probiotic Yeast *Saccharomyces boulardii*

**DOI:** 10.1371/journal.pone.0112660

**Published:** 2014-11-12

**Authors:** Lauren E. Hudson, Milo B. Fasken, Courtney D. McDermott, Shonna M. McBride, Emily G. Kuiper, David B. Guiliano, Anita H. Corbett, Tracey J. Lamb

**Affiliations:** 1 Department of Pediatrics, Emory University School of Medicine, Atlanta, Georgia, United States of America; 2 Department of Biochemistry, Emory University School of Medicine, Atlanta, Georgia, United States of America; 3 Department of Microbiology and Immunology, Emory University School of Medicine, Atlanta, Georgia, United States of America; 4 School of Health, Sport and Bioscience, University of East London, London, United Kingdom; CNR, Italy

## Abstract

Recent studies have suggested the potential of probiotic organisms to be adapted for the synthesis and delivery of oral therapeutics. The probiotic yeast *Saccharomyces boulardii* would be especially well suited for this purpose due to its ability, in contrast to probiotic prokaryotes, to perform eukaryotic post translational modifications. This probiotic yeast thus has the potential to express a broad array of therapeutic proteins. Currently, however, use of wild type (WT) *S. boulardii* relies on antibiotic resistance for the selection of transformed yeast. Here we report the creation of auxotrophic mutant strains of *S. boulardii* that can be selected without antibiotics and demonstrate that these yeast can express functional recombinant protein even when recovered from gastrointestinal immune tissues in mice. A UV mutagenesis approach was employed to generate three uracil auxotrophic *S. boulardii* mutants that show a low rate of reversion to wild type growth. These mutants can express recombinant protein and are resistant *in vitro* to low pH, bile acid salts, and anaerobic conditions. Critically, oral gavage experiments using C57BL/6 mice demonstrate that mutant *S. boulardii* survive and are taken up into gastrointestinal immune tissues on a similar level as WT *S. boulardii*. Mutant yeast recovered from gastrointestinal immune tissues furthermore retain expression of functional recombinant protein. These data show that auxotrophic mutant *S. boulardii* can safely express recombinant protein without antibiotic selection and can deliver recombinant protein to gastrointestinal immune tissues. These auxotrophic mutants of *S. boulardii* pave the way for future experiments to test the ability of *S. boulardii* to deliver therapeutics and mediate protection against gastrointestinal disorders.

## Introduction

*Saccharomyces cerevisiae* subspecies *boulardii* is a generally recognized as safe (GRAS) yeast strain classified as a subspecies of the well characterized laboratory yeast *S. cerevisiae* [1], [2]. *S. boulardii* is currently used as a probiotic to treat antibiotic-induced diarrhea in children and adults, recurrent *Clostridium difficile* infections, inflammatory bowel disease, and other gastrointestinal disorders [3], [4]. The exact mechanisms by which *S. boulardii* mediates these protective effects are not fully understood. However, administration of *S. boulardii* in animal models has been shown to increase secretory IgA, interleukin 10 (IL-10), and IL-10 induced T regulatory cells [5], [6] as well as to preserve intestinal epithelial integrity in colitis models [7]–[9] and to degrade specific pathogen toxins [10], [11].

Key features of *S. boulardii* have raised the interesting prospect of using this probiotic yeast not only as a preparation of wild type cells for the treatment of gastrointestinal disorders, but also as a vehicle for drug synthesis and delivery to the intestine. First, targeted delivery of drug to the gastrointestinal tract could permit lower drug doses relative to systemic administration as well as facilitate more direct interactions with the mucosal immune system. Second, genetically modified yeast would be a less expensive alternative to many proposed delivery mechanisms, such as nanoparticles and liposomes, as yeast can be economically produced on a large, industrial scale. Indeed, *S. cerevisiae* is already used to produce such compounds as insulin, hepatitis B surface antigen, granulocyte macrophage colony stimulating factor (GM-CSF), and platelet derived growth factor (reviewed in [12]).

*S. boulardii* also has several advantages relative to other live microorganisms proposed as drug delivery vehicles. As a eukaryotic organism capable of expressing complex, glycosylated antigens, *S. boulardii* can potentially express a much wider array of compounds than probiotic bacteria. Also, *S. boulardii* shows increased resistance to higher temperatures and low pH relative to conventional laboratory strains of *S. cerevisiae* [13], [14], which could translate to an increased ability of *S. boulardii* to survive transit through the intestine. Furthermore, *S. boulardii* is not a natural colonizer of the gastrointestinal tract in humans or mice [15]–[18], which would allow for accurate drug dosing given reliable clearance of *S. boulardii* from the intestine.

Although transformation of DNA into *S. boulardii* has been reported to be less efficient than transformation of DNA into *S. cerevisiae* [19], various methods of transformation have recently been evaluated in *S. boulardii* [20]. Several studies have reported successful transformation of DNA and production of recombinant protein in *S. boulardii* [13], [19], [21]–[23]; however, feasibility of this application is currently limited because prototrophic, wild type (WT) *S. boulardii* can be transformed and selected only with antibiotic resistance markers. Clinical use of these transformed yeast on a large scale would thus carry risk of transferring antibiotic resistance markers to the microbiota. A common alternative to antibiotic selection of transformed yeast is the use of auxotrophic mutants. Auxotrophic yeast lack enzymes critical for the synthesis of essential amino acids or pyrimidines and can grow in selective media only if they are transformed with a plasmid encoding the required enzyme. Unfortunately, the only existing *S. boulardii* auxotroph is unavailable for use in the United States [22]. Thus there remains a need to generate an auxotrophic strain of *S. boulardii* that can be easily manipulated without the use of antibiotic resistance markers. This auxotrophic strain would also need to produce recombinant protein during transit through the gut despite the harsh digestive conditions and lack of selective pressure. Such an auxotrophic strain would make *S. boulardii* a much safer and more efficient vehicle to express and deliver recombinant proteins to treat gastrointestinal disorders.

To develop a strain of *S. boulardii* that can be transformed without antibiotic selection markers, we used a UV mutagenesis approach and selected for auxotrophic mutants that lack a functional orotidine 5′-phosphate decarboxylase (Ura3), encoded by the *URA3* gene. The Ura3 enzyme decarboxylates orotidine monophosphate (OMP) to form uridine monophosphate (UMP) in the *de novo* synthesis pathway of pyrimidines. The *ura3^−^* auxotrophic yeast generated are unable to grow on media lacking uracil, allowing for positive selection of *ura3^−^* mutant yeast transformed with a *URA3* plasmid on media lacking uracil. In addition, Ura3 converts the compound 5-fluoroorotic acid (5-FOA) to the toxin 5-fluorouracil, inducing cellular death of *URA3* ^+^ yeast plated on media containing 5-FOA and allowing for easy identification of *ura3^−^* colonies.

Here we employed UV mutagenesis and 5-FOA screening to generate three *S. boulardii ura3^−^* auxotrophic mutants. These mutants can be transformed and selected without the use of antibiotics. Furthermore, these mutants maintain the resistance to bile acid and low pH that is characteristic of WT *S. boulardii* and are taken up into immune tissues of the murine gastrointestinal tract at a frequency similar to that of WT *S. boulardii*. These mutant yeast also continue to express functional recombinant protein after passage through the intestine and uptake into immune tissues. In sum, we have developed *S. boulardii* strains that could be adapted for use in the synthesis and delivery of drug to the gastrointestinal tract.

## Materials and Methods

### Chemicals and Plasmids

All chemicals were obtained from Sigma, U. S. Biological Corp. or Fisher Scientific unless otherwise noted, and all media were prepared according to standard procedures [24]. All DNA manipulations were performed according to standard methods [25]. To construct the plasmids used in transformation of *S. boulardii* mutants, the constitutive yeast promoter *TEF1* from the p427 plasmid (Dualsystems Biotech AG) was subcloned into pRS426 [26], a 2μ plasmid containing the *S. cerevisiae URA3* sequence. The coding sequence of GFP was then subcloned into the pRS426 plasmid under the *TEF1* promoter.

### Yeast Strains

Table 1 lists all yeast strains used, their auxotrophic markers, and sources. WT *S. boulardii* (Ultra Levure, American Type Culture Collection Number: MYA-797) was used for all mutagenesis experiments. UV sensitive *S. cerevisiae rad1* (SND 713) [27] was used as a positive control in UV irradiation experiments. *S. cerevisiae RAD1* (SND 711) [27] is a wild type strain isogenic to *S. cerevisiae rad1* and was used for comparison in generating UV survival curves. *S. cerevisiae* RM11-1a (GCY 2860), a wild type haploid strain [28], was used in pH and bile acid testing. *S. cerevisiae* W303, a well characterized laboratory haploid strain (http://yeastgenome.org/), was used in pH, bile acid, and anaerobic testing. *S. cerevisiae* YH990, a diploid strain, was used for comparison in survival curve generation as well as pH, bile acid, and anaerobic testing [29].

**Table 1:** Tinoofpression by aromyces boulardii Auxotrophicine ii to create an optimal probiotic drug delivery system.

Strain	Designation	Description	Source
*S. boulardii*	WT *S. boulardii*	MYA-797	American Type Culture Collection
*S. cerevisiae* SND 713	*S. cerevisiae rad1*	*rad1*::kanMX; spore of hNDP223	[27]
*S. cerevisiae* SND 711	*S. cerevisiae RAD1*	WT spore of hNDP223	[27]
*S. cerevisiae* W303	*S. cerevisiae* laboratory haploid	*MATα ura3*Δ* leu2*Δ* trp1*Δ* his3*Δ	http://www.yeastgenome.org/
*S. cerevisiae* YH990	*S. cerevisiae* diploid	2n a/α by YEpHO-LEU2 of E134	[29]
*S. cerevisiae* RM11-1a (GCY 2860)	*S. cerevisiae* wild type haploid	*MATα ho::loxP lys2*Δ*0 ura3*Δ*0*	Generated by transient Cre expression to eliminate G418 resistance marker in strain UCC1159 [28]

### Survival Curve Generation

All media used for yeast growth were prepared according to standard procedures [24]. Overnight YPD (1% yeast extract, 2% peptone, 2% glucose/dextrose in distilled water) cultures of WT *S. boulardii* and three *S. cerevisiae* strains (WT diploid, *rad1*, and *RAD1*) were resuspended in 20 mL distilled water at a concentration of 10^7^ cells/mL. Diluted cells were then placed in a sterile plastic petri dish 14 cm below the UV bulb of an Eppendorf UV Stratalinker. Cells were exposed to UV irradiation with the lid removed, with 500 µL of cells extracted at increments of 0 µJ, 5,000 µJ, 10,000 µJ, 15,000 µJ, 20,000 µJ, 22,500 µJ, 25,000 µJ, 30,000 µJ, and 50,000 µJ. Irradiated cells were serially diluted and plated onto YPD media (2% agar, 1% yeast extract, 2% peptone, 2% glucose/dextrose in distilled water). Plates were covered in aluminum foil to prevent photo-reactivation, and colony forming units (CFU) were counted after 3–5 days incubation at 30°C.

### Screening of UV Irradiated Cells

WT *S. boulardii* was prepared and irradiated as for generation of survival curves with *S. cerevisiae rad1* used as a control. Cells were given doses of UV irradiation corresponding to approximately 50% WT *S. boulardii* survival (20,000–22,500 µJ) and plated onto media containing 5-fluoroorotic acid (5-FOA) to select for cells lacking a functional *URA3* gene [30], [31]. Colonies resistant to 5-FOA were screened by multiple restreaking onto new plates containing 5-FOA and plates lacking uracil as well as by assessing growth in liquid media lacking uracil.

### Confirmation of URA3 mutations

The primers URA3_Fwd (CCTGCAGGAAACGAAGATAAATCATGTCGAAAGCTACATA) and URA3_Rev (CATTTACTTATAATACAGTTTTTTAGTTTTGCTGGCCGCA) were used to PCR amplify the 804 bp *URA3* coding region of the *S. boulardii* mutants. PCR products were purified using the PCR Purification Kit (Qiagen) and submitted to Beckman Coulter Genomics for sequencing.

### pH and Bile Acid Testing

WT *S. boulardii*; *S. boulardii ura3^−^* Mutants 1, 2, and 3; and *S. cerevisiae* laboratory haploid (W303), diploid (YH990), and WT haploid (Rm11-1a) strains were grown overnight in YPD. Cells (5×10^7^) were then resuspended in 500 µL YPD (approximately pH 6); YPD adjusted to pH 2, 4, or 8 via addition of either 12 N HCL or NaOH; complete media containing 0.3% OxGall (US Biologicals); or media lacking uracil. For each dilution, 100 µL was aliquoted in duplicate in 96 well plates (10^7^ cells per well), and optical density 600 (OD_600_) readings were taken over 24 hour incubation at 37°C using a Bio Tek Instruments ELx 808 Ultra Microplate Reader to assess growth.

### Anaerobic Testing

WT *S. boulardii*; *S. boulardii ura3^−^* Mutants 1, 2, and 3; and *S. cerevisiae* laboratory haploid (W303), diploid (YH990), and WT haploid (Rm11-1a) strains were grown overnight in YPD. Yeast were diluted to 5×10^7^ cells/mL in fresh YPD and incubated in a vinyl anaerobic chamber (Type B; Coy Laboratory Products) maintained at 37°C. The atmosphere of the chamber was filled with an anaerobic gas mix comprised of 85% nitrogen, 10% hydrogen and 5% carbon dioxide, and was set up and operated as previously described [32]. One milliliter samples were taken over 24 hours to measure OD_600_ values. Samples were also taken at 12 and 24 hours for CFU counts.

### Yeast Transformation

All yeast were transformed using standard electroporation [33] and LiOAc [24] protocols. Briefly, for LiOAc transformation, overnight cultures were diluted to 2×10^6^ cells/mL in fresh YPD and incubated at 30°C until reaching a concentration of 10^7^cells/mL. Cells were then washed in sterile water and TE/LiOAc and combined with plasmid DNA, carrier DNA, and PEG/TE/LiOAc and agitated for 30 minutes at 30°C. DMSO was then added and cells were heat shocked at 42°C for 15 minutes, washed, and plated onto selective media. For electroporation, cells were grown overnight to saturation, diluted to an OD_600_ of 0.2 and incubated until reaching an OD_600_ of 1.6. Cells were washed with ice cold water and buffer containing 1 M sorbitol and 1 mM CaCl_2_. Cells were resuspended in 100 mM LiOAc/10 mM DTT and agitated for 30 min at 30°C. Pelleted cells were then washed and resuspended in buffer containing 1 M sorbitol and 1 mM CaCl_2_. A 400 µL volume of cells was combined with DNA, then electroporated using a BioRad micropulser. Cells were then transferred to a 1∶1 mixture of YPD and 1 M sorbitol and incubated one hour at 30°C with agitation. Cells were plated onto media containing 1 M sorbitol.

### Analysis of GFP Fluorescence

Images of untransformed yeast and yeast transformed with a *URA3* plasmid encoding GFP were collected using an Olympus IX80. Flow cytometry was performed by resuspending cells in FACS buffer (sterile PBS and 0.5% FBS) and analyzing them using a BD LSR II flow cytometer and B530/30 filter.

### Isolation of Viable Yeast from Murine Peyer's Patches

All animal experiments were conducted strictly in adherence to the guidelines and recommendations in the National Institutes of Health Guide for the Care and Use of Laboratory Animals. Experiments were approved by the Emory University Institutional Animal Care and Use Committee (Protocol number: DAR-2002655-021817BN), and euthanasia was performed using CO_2_. WT female C57BL/6J mice aged 6–8 weeks were gavaged 10^8^ CFU of either WT *S. boulardii*, *S. cerevisiae* laboratory haploid, or *S. boulardii* Mutant 2. Peyer's patches from each mouse were harvested four hours post gavage, cell strained, and plated onto yeast media to detect viable colonies. YPD plates were used in plating of untransformed yeast and plates lacking uracil were used to select *S. cerevisiae* laboratory haploid and *S. boulardii* Mutant 2 transformed with a *URA3* plasmid. CFU were counted after 2–4 days incubation at 30°C. The sample size needed to determine a statistically significant difference was calculated using Lehr's formula n = 2(1.96+0.8416)^2^/(d/s)^2^ where d is the smallest meaningful difference in means and s is the standard deviation of the observations in each group, assuming a power of 80% and a significance of 5% [34].

## Results

Numerous studies have characterized superior growth of *S. boulardii* relative to *S. cerevisiae* strains [13], [14]. In order to test growth of *S. boulardii* (ATCC MYA-797) compared to the *S. cerevisiae* strains used in this study (Table 1), yeast were incubated for 24 hours at either 30°C, the optimal growth temperature for most *S. cerevisiae* strains [20], or 37°C, normal human body temperature. Growth of *S. boulardii* was compared to three strains of *S. cerevisiae*. W303 was selected due its frequent use as an *S. cerevisiae* laboratory haploid strain. RM11-1a, an *S. cerevisiae* WT haploid strain that has been more recently isolated and which carries a lower rate of age related mutations relative to other *S. cerevisiae* strains, was used as a natural isolate comparison to *S. boulardii* [28]. Finally, *S. cerevisiae* YH990, a diploid, was used to compare growth of *S. boulardii* to that of another diploid yeast strain.

**Figure 1:**
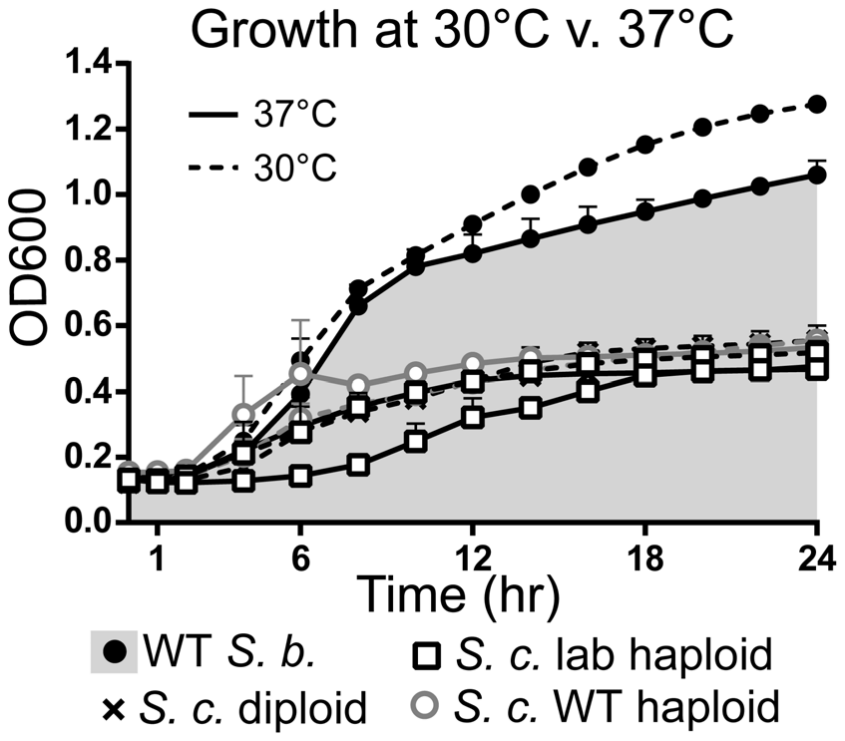
*S. boulardii* Shows Enhanced Growth Relative to *S. cerevisiae* at Both 30°C and 37°C. Yeast were grown overnight in YPD and diluted to 10^7^ cells per well in a 96 well plate. OD_600_ readings over 24 hours incubation at 37°C or 30°C indicate relative growth of wild type *S. boulardii* (WT *S.b.*), *S. cerevisiae* laboratory haploid (*S.c.* lab haploid), *S. cerevisiae* wild type haploid (*S.c.* WT haploid), and *S. cerevisiae* diploid (*S.c.* diploid). Lines represent the mean of duplicate experiments, with error bars depicting plus the standard error of the mean (SEM). Shading highlights growth of yeast strains relative to growth of W T *S. boulardii* at 37°C.

As shown in Figure 1, *S. boulardii* shows a faster rate of growth and higher saturation point at both 37°C and 30°C relative to all three *S. cerevisiae* strains tested (laboratory haploid, WT haploid, and diploid) in normal rich media (YPD). Although *S. boulardii* actually reaches a higher saturation point at 30°C versus 37°C, its superior growth at 37°C relative to *S. cerevisiae* indicates that *S. boulardii* is more likely to show better growth at body temperature than *S. cerevisiae*.

### Diploid *S. boulardii* Require High Doses of UV Irradiation to Achieve 50% Cell Survival

UV mutagenesis coupled with 5-FOA resistance was used to screen for *ura3^−^ S. boulardii* mutants. Previous UV mutagenesis studies have used high UV doses, resulting in only 10–20% survival, to screen for auxotrophic mutants [22], [35]. Most of these studies targeted haploid *S. cerevisiae* strains; however, there has been only one report of tetrad formation and isolation of haploid *S. boulardii* cells [13]. Indeed, attempts in the present study to induce *S. boulardii* sporulation were unsuccessful. Higher doses of UV irradiation may thus be necessary to increase the likelihood of inducing homozygous mutations in both copies of *S. boulardii URA3*. However, in light of potential future *in vivo* applications, lower doses of irradiation would be optimal to avoid mutating genes related to *S. boulardii*'s superior growth and immunomodulatory characteristics. A 50% survival dose of UV irradiation was therefore chosen to screen for *ura3* ^−^ *S. boulardii* mutants.

**Figure 2:**
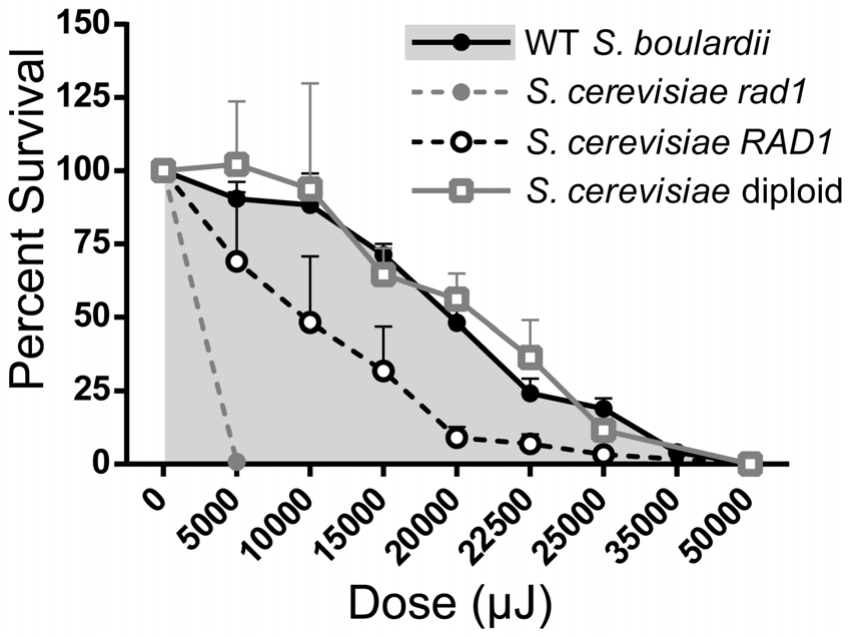
Fifty Percent of *S. boulardii* Cells Survive at 20,000–22,500 µJ UV Irradiation. Wild type (WT) *S. boulardii* and *S. cerevisiae* diploid, haploid *RAD1*, and haploid *rad1* were exposed to various doses of UV irradiation. Percent survival (CFU as a percentage of total cells irradiated and plated) was plotted at each dose (mean of n = 2 per strain per UV dose, with error bars depicting plus the standard error of the mean) to identify the dose of UV irradiation corresponding to 50% survival of WT *S. boulardii* cells. Greater than 100% survival was likely reached at some low UV doses due to cellular replication after irradiation.

To determine the UV dose necessary to kill 50% of *S. boulardii* cells, WT *S. boulardii* as well as *S. cerevisiae* strains laboratory haploid, diploid, *RAD1*, and UV sensitive *rad1* cells were exposed to UV irradiation (Fig. 2). As expected, the *S. cerevisiae rad1* mutant was killed even with low doses (5,000 µJ) of UV irradiation. Comparing percent survival versus UV dose shows that, as expected, higher doses of UV irradiation are needed to kill WT *S. boulardii* and *S. cerevisiae* diploid cells relative to *S. cerevisiae* haploid *RAD1* cells. The UV dose corresponding to approximately 50% WT *S. boulardii* survival was determined to be 20,000–22,500 µJ and was used for subsequent screening for *S. boulardii ura3^−^* mutants.

### Isolation of Three *S. boulardii* Mutants Unable to Grow Without Uracil

**Figure 3:**
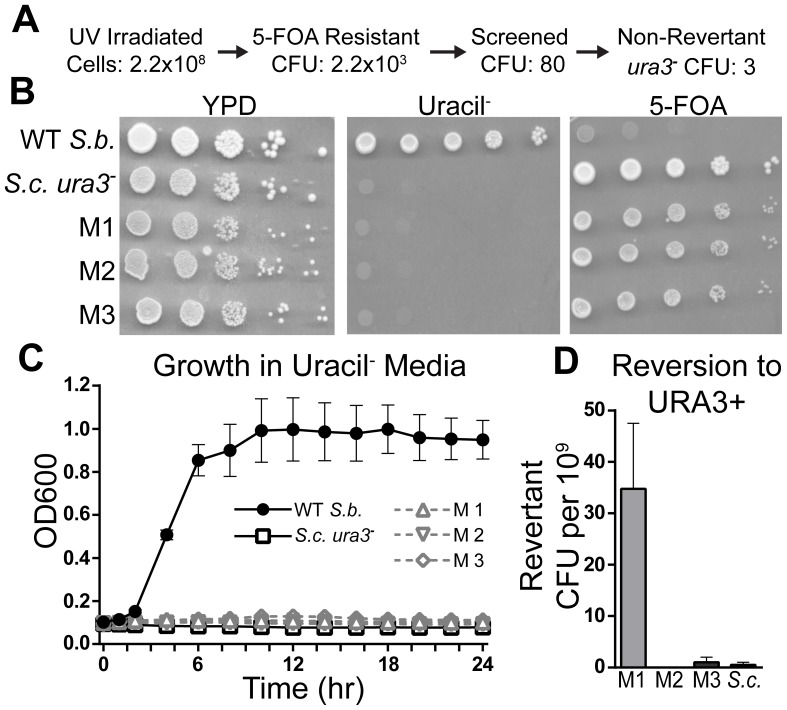
Isolation of Three *S. boulardii* Mutants Unable to Grow Without Uracil. (A) Flow diagram depicting the number of irradiated wild type (WT) *S. boulardii* cells, screened 5-FOA resistant colonies, and final number of *S. boulardii ura3* ^−^ mutants obtained. (B) Growth of WT *S. boulardii*, *S. boulardii* Mutants 1–3 (M1, M2, M3), and *ura3* ^−^ *S. cerevisiae* laboratory haploid was assessed by serial dilution and spotting on YPD, uracil^−^, and 5-FOA plates. (C) Growth of *S. boulardii* Mutants 1–3 relative to WT *S. boulardii* and *ura3* ^−^ *S. cerevisiae* laboratory haploid at 37°C in liquid media lacking uracil. Lines represent the mean of duplicate experiments for each strain, with error bars depicting plus and minus the standard error of the mean (SEM). (D) Number of CFU able to grow on plates lacking uracil per 10^9^ plated cells. Each bar depicts the mean of duplicate experiments with error bars depicting plus the SEM.

Approximately 2.2×10^8^ WT *S. boulardii* cells were irradiated at a 50% survival dose (20,000–22,500 µJ) and plated onto media containing 5-FOA (Fig. 3A). Of these irradiated cells, approximately 2,200 were 5-FOA resistant. Eighty of these 5-FOA resistant colonies were further screened to confirm their ability to grow on plates containing 5-FOA and their inability to grow on plates or in liquid media lacking uracil (Fig. 3B, C). As expected, WT *S. boulardii* can grow on YPD plates or plates lacking uracil but does not grow on plates containing 5-FOA. In contrast, *S. boulardii* Mutants 1, 2, and 3 (M1, M2, and M3) show a pattern of growth similar to that of the laboratory haploid *ura3^−^ S. cerevisiae* strain, with the ability to grow on YPD plates or plates containing 5-FOA, but not on plates lacking uracil. Although the vast majority of colonies originally isolated from 5-FOA plates showed a high rate of reversion to a *URA3^+^* phenotype (approximately 1–2% reversion), *S. boulardii* Mutants 1–3 showed a relatively low rate of reversion (Fig. 3D) comparable to that seen for a commonly used *ura3* ^−^ laboratory haploid *S. cerevisiae* strain. Indeed, *S. boulardii* M2 showed no detectable reversion.

**Figure 4:**
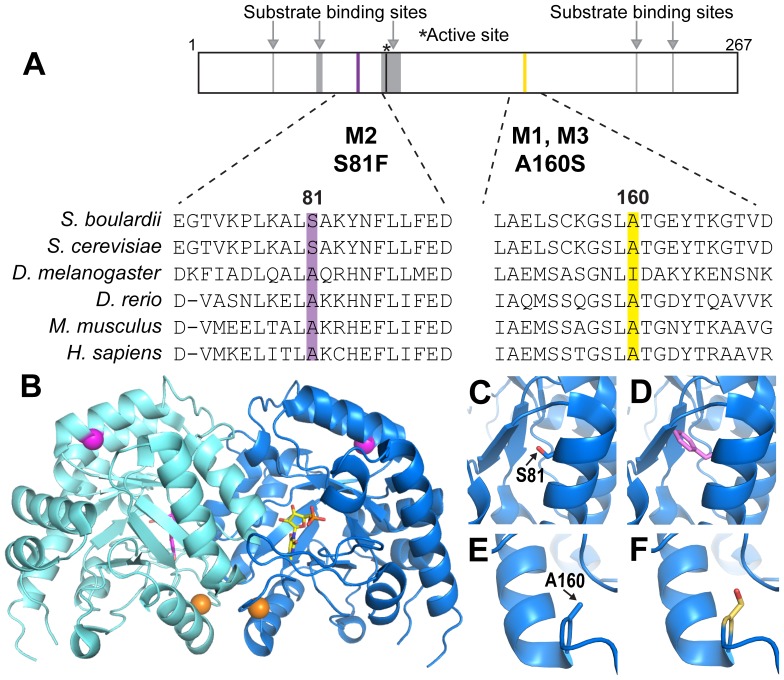
*S. boulardii ura3* ^−^ Mutants Contain Single Amino Acid Changes Within the Ura3 Protein. (A) Schematic showing the domain structure of Ura3 protein in regions surrounding the amino acid changes in *S. boulardii ura3* ^−^ mutants. Ura3 substrate binding sites are shown in gray with arrows above (amino acids 37, 59–61, 91–100, 217, 235) and the active site as a black line with asterisk above (amino acid 93). The altered amino acid sites in the *S. boulardii* mutants are shown as purple (S81F in M2) and yellow (A160S in M1 and M3) lines with the changes indicated below. Homologous regions including the altered amino acids and the 20 surrounding residues in *Homo sapiens*, *Mus musculus*, *Danio rerio*, *Drosophila melanogaster*, *Saccharomyces cerevisiae*, and WT *S. boulardii* are depicted to show conservation of these residues. (B) Ribbon depiction of the *S. cerevisiae* Ura3 homodimer bound to the proposed transition state analog 6-hydroxyuridine 5′-phosphate (PDB ID: 1DQX) [36]. The *S. boulardii* mutant single amino acid changes are noted in yellow (A160S in M1 and M3) and purple (S81F in M2). (C) Enlarged view showing the wild type serine residue at position 81. (D) Enlarged view showing the amino acid change to phenylalanine at position 81 in *S. boulardii* Mutant 2. (E) Enlarged view showing the wild type alanine residue at position 160. (F) Enlarged view showing the amino acid change to serine at position 160 in *S. boulardii* Mutants 1 and 3.

Sequence analysis of the *URA3* open reading frame in *S. boulardii* Mutants 1–3 revealed single amino acid substitutions located outside of specific functional domains (Fig. 4A). M1 and M3 both contained an A160S amino acid substitution, while M2 contained an S81F amino acid substitution. Although simultaneous mutation of both copies of *URA3* in the diploid *S. boulardii* is likely to be an extremely rare event, selective pressure due to the presence of 5-FOA could have facilitated duplication of mutated *URA3*. Structural modeling of the *S. cerevisiae* Ura3 protein (PDB ID: 1DQX [36]) (Fig. 4B, C) reveals that residue serine 81 is located within an α-helix. A change from serine (Fig. 4C) to the larger phenylalanine (Fig. 4D) at residue 81 could cause a steric clash with surrounding amino acids including phenylalanine 86 and leucine 87 on the opposing β strand, likely impairing proper protein folding and catalytic function. The reason for lack of Ura3 function in M1 and M3 is less clear than for M2. Residue 160 is located approximately 10 Å from the catalytic site and outside of any α-helices or β-pleated sheets (Fig. 4B, E–F). Although residue 160 is conserved in *Homo sapiens*, *Mus musculus*, *Danio rerio*, and WT *S. boulardii* (Fig. 4A) as alanine, the *S. cerevisiae* +D4 Ura3 protein contains a serine at the homologous position [37]. Furthermore, the crystal structure of Ura3 from *S. cerevisiae* has been solved with an A160S substitution (Fig. 4B) (PDB ID: 1DQX [36]). These data suggests that mutations outside the open reading frame, such as in promoter or enhancer regions, might instead account for lack of Ura3 function in M1 and M3.

### *S. boulardii* Mutants are Resistant to Low pH and Bile Acid *In Vitro*

**Figure 5:**
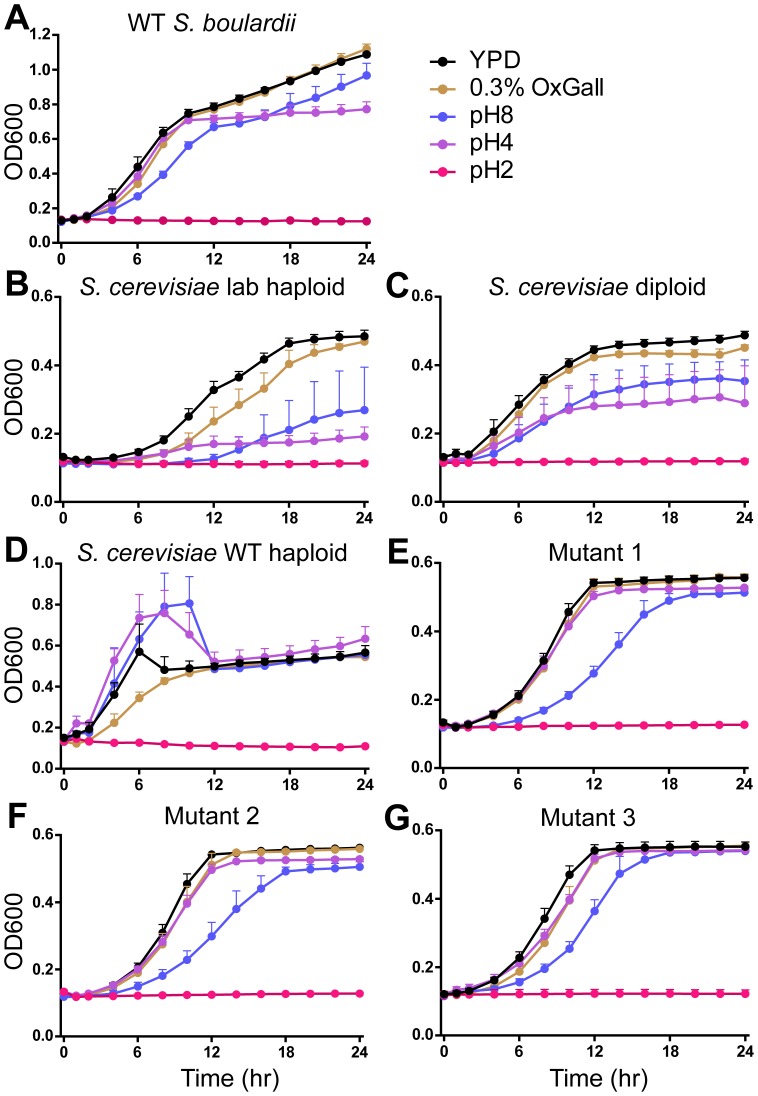
*S. boulardii ura3* ^−^ Mutants are Resistant to *In Vitro* Intestine-Like Conditions. Yeast were grown overnight in YPD and diluted to 10^7^ cells per well in a 96 well plate. OD_600_ readings were taken over 24 hours incubation at 37°C. Graphs depict growth of yeast strains at pH 2, pH 4, pH 8, 0.3% OxGall, and YPD (approximately pH 6). Yeast strains include wild type (WT) *S. boulardii* (A); *S. cerevisiae* strains laboratory haploid (B), diploid (C), and wild type haploid (D); and *S. boulardii* M1 (E), M2 (F), and M3 (G). This analysis shows that *S. boulardii* mutants maintain resistance to pH 4 and pH 8 as well as to 0.3% OxGall whereas *S. cerevisiae* strains laboratory haploid and diploid are sensitive to these conditions.

As in previous studies [13], [14], *S. boulardii* shows enhanced growth relative to *S. cerevisiae* strains in YPD as well as in media at pH 4, pH 8, and containing bile salts (0.3% OxGall) (Fig. 5 A–D). In order to determine if *S. boulardii* Mutants 1–3 retained the characteristic ability of WT *S. boulardii* to withstand pH changes and bile acid, WT and mutant *S. boulardii* were grown in pH-adjusted media for 24 hours (Fig. 5A, E–G). All three *S. boulardii* mutants grow similarly in media at pH 4, pH 8, and 0.3% OxGall and reach a similar optical density (OD_600_) at saturation. Notably, growth of all three *S. boulardii* mutants in YPD is decreased relative to WT *S. boulardii* (OD_600_ of approximately 0.6 at saturation for *S. boulardii* mutants versus over 1.0 for WT *S. boulardii*) (compare Fig. 5A to Fig. 5E–G). However, growth of *S. boulardii* mutants in media containing 0.3% OxGall or media at pH 4 is decreased only slightly relative to mutant growth in YPD. Furthermore, *S. boulardii* mutants appear less affected by media at pH 8 relative to *S. cerevisiae* laboratory haploid and diploid cells. Although the *S. boulardii* mutant growth rate at pH 8 is decreased relative to growth in YPD, OD_600_ at saturation in media at pH 8 almost reaches that seen with mutant growth in YPD. In contrast, growth of *S. cerevisiae* laboratory haploid and diploid strains at pH 8 never reaches that seen in YPD. These results indicate that while the saturation point for growth of *S. boulardii* mutants is decreased relative to WT *S. boulardii*, pathways influencing pH and bile acid resistance have been maintained.

### *S. boulardii* Mutants Show Increased Growth in Anaerobic Conditions

**Figure 6:**
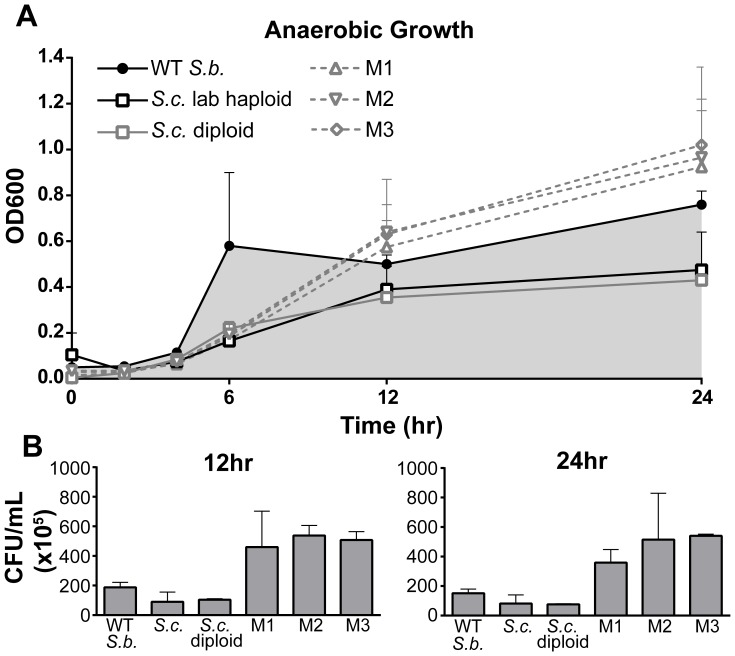
*S. boulardii ura3* ^−^ Mutants Grow in *In Vitro* Anaerobic Conditions. (A) Wild type (WT) *S. boulardii*; *S. cerevisiae* strains laboratory haploid, diploid, and wild type haploid; and *S. boulardii* M1, M2, and M3 were grown overnight in YPD and diluted to 5×10^7^ cells/mL in fresh YPD. OD_600_ readings were taken over 24 hours incubation in a vinyl anaerobic chamber maintained at 37°C. (B) Number of colony forming units (CFU) per mL for each yeast strain after 12 and 24 hours incubation in the vinyl anaerobic chamber. This analysis shows that WT *S. boulardii* and particularly *S. boulardii* Mutants 1–3 show superior growth in anaerobic conditions relative to *S. cerevisiae* strains.

Given the ability of WT *S. boulardii* to grow in the anaerobic conditions of the gastrointestinal system, WT *S. boulardii* and Mutants 1–3 were incubated in anaerobic conditions for 24 hours (Fig. 6). OD_600_ readings show that both WT and mutant *S. boulardii* grow more quickly and to a higher saturation point than the tested *S. cerevisiae* strains (lab haploid and diploid) (Fig. 6A). Similarly, CFU counts of samples taken at 12 and 24 hours incubation in anaerobic conditions show the highest number of viable cells for Mutants 1–3 followed by WT *S. boulardii*, with the lowest cell numbers for *S. cerevisiae* lab haploid and diploid (Fig. 6B).

### *S. boulardii* Mutants Can Be Transformed and Express Functional GFP

**Figure 7:**
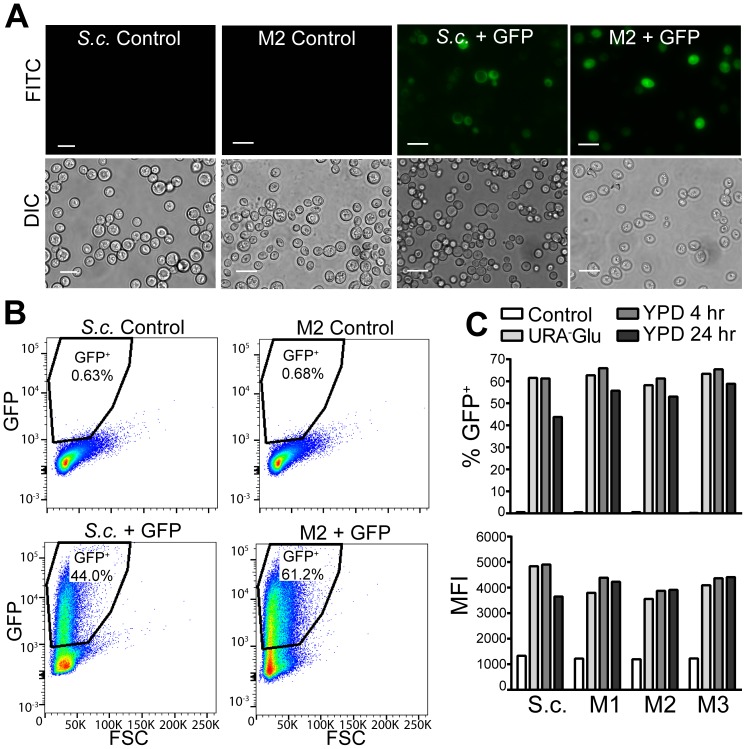
*S. boulardii* Mutants Express Functional GFP. (A) Bright field and fluorescent images of *ura3^−^ S. cerevisiae* laboratory haploid (*S.c.*) and *S. boulardii* Mutant 2 (M2) either untransformed (Control) or transformed (+GFP) with a *URA3* plasmid containing GFP. The GFP fluorescence is detected in the FITC channel. Corresponding differential interference contrast (DIC) images are also shown. Scale bars show 10 µm. (B) Representative flow cytometry plots of forward-scattered light (FSC) versus GFP fluorescence for untransformed (Control) and transformed (+GFP) *S. cerevisiae* laboratory haploid (*S.c.* lab haploid) and *S. boulardii* Mutant 2 (M2) showing the percent of GFP positive cells in each population (n = 2 per strain). Transformed yeast were maintained in media lacking uracil prior to analysis. (C) Retention of *URA3* plasmid and GFP expression was tested by comparing the percent of GFP positive cells of untransformed yeast (Control) relative to transformed yeast cultured in either selective media lacking uracil (URA^−^Glu), YPD (non selective media) for 4 hours (YPD 4 hr), or YPD for 24 hours (YPD 24 hr). Yeast strains analyzed include untransformed and transformed *ura3^−^ S. cerevisiae* laboratory haploid (*S.c.*) and *S. boulardii* Mutants 1–3 (M1, M2, M3). Median fluorescent intensity (MFI) of GFP positive cells in each population is also depicted, indicating there is no visible decrease in average GFP expression per cell after incubation in YPD for 4 or 24 hours. Bars depict the mean of two samples per strain per incubation condition.

In order to determine whether the *S. boulardii* mutants can be successfully transformed and express heterologous protein, *S. boulardii* Mutants 1–3 were transformed with a *URA3* plasmid encoding GFP. Fluorescence microscopy reveals GFP fluorescence in transformed *S. boulardii* mutants and *S. cerevisiae* laboratory haploid cells, but no background fluorescence in untransformed yeast (Fig. 7A). In addition, flow cytometry analysis shows a high percentage of GFP-expressing cells in transformed *S. cerevisiae* (44% v. 0.63% for transformed and untransformed yeast, respectively) and *S. boulardii* Mutant 2 (61.2% v. 0.68% for transformed and untransformed yeast, respectively) (Fig. 7B). These results demonstrate that *S. boulardii* mutants can express GFP as efficiently as the well characterized *S. cerevisiae* laboratory haploid strain.

To test the ability of *S. boulardii* mutants to maintain plasmid and heterologous protein expression without selective pressure, as will occur in the gastrointestinal system, transformed yeast were incubated in YPD for 4 or 24 hours and subsequently tested for GFP expression. As shown by flow cytometry, transformed *S. cerevisiae* and *S. boulardii* Mutants 1–3 incubated in YPD for 4 or 24 hours maintained a high percentage of GFP-expressing cells comparable to that of yeast maintained in selective media lacking uracil (Fig. 7C). GFP positive *S. boulardii* mutant cells also maintained comparable median fluorescence intensity after incubation in non selective YPD media, indicating that on average not only the number of cells but also GFP expression per cell was maintained over 24 hours without selective pressure.

### Viable Transformed *S. boulardii* Mutant 2 Expressing GFP Can Be Isolated from Murine Peyer's Patches

Use of transformed mutant *S. boulardii* for delivery of recombinant protein to the intestine depends not only on the ability to maintain plasmid without selection but also on the ability to survive passage through the gastrointestinal tract. Furthermore, in the case of cytokine delivery, the ability to contact immune tissues of the small intestine will be critical in helping to induce anti-inflammatory responses. Peyer's patches are major sites of antigen sampling from the small intestine lumen as well as key sites of immune response induction and development [38]. Thus, uptake of transformed yeast into Peyer's patches would indicate the ability of yeast not only to survive passage through the gastrointestinal tract but also to contact tissues responsible for mediating immune responses. As all three *S. boulardii* mutants showed similar resistance *in vitro* to low pH and bile acid (Fig. 5E–G) and to anaerobic conditions (Fig. 6), *S. boulardii* Mutant 2 was used for *in vivo* experiments in mice as this mutant has no detectable reversion to a *URA3^+^* phenotype (Fig. 3D).

**Figure 8:**
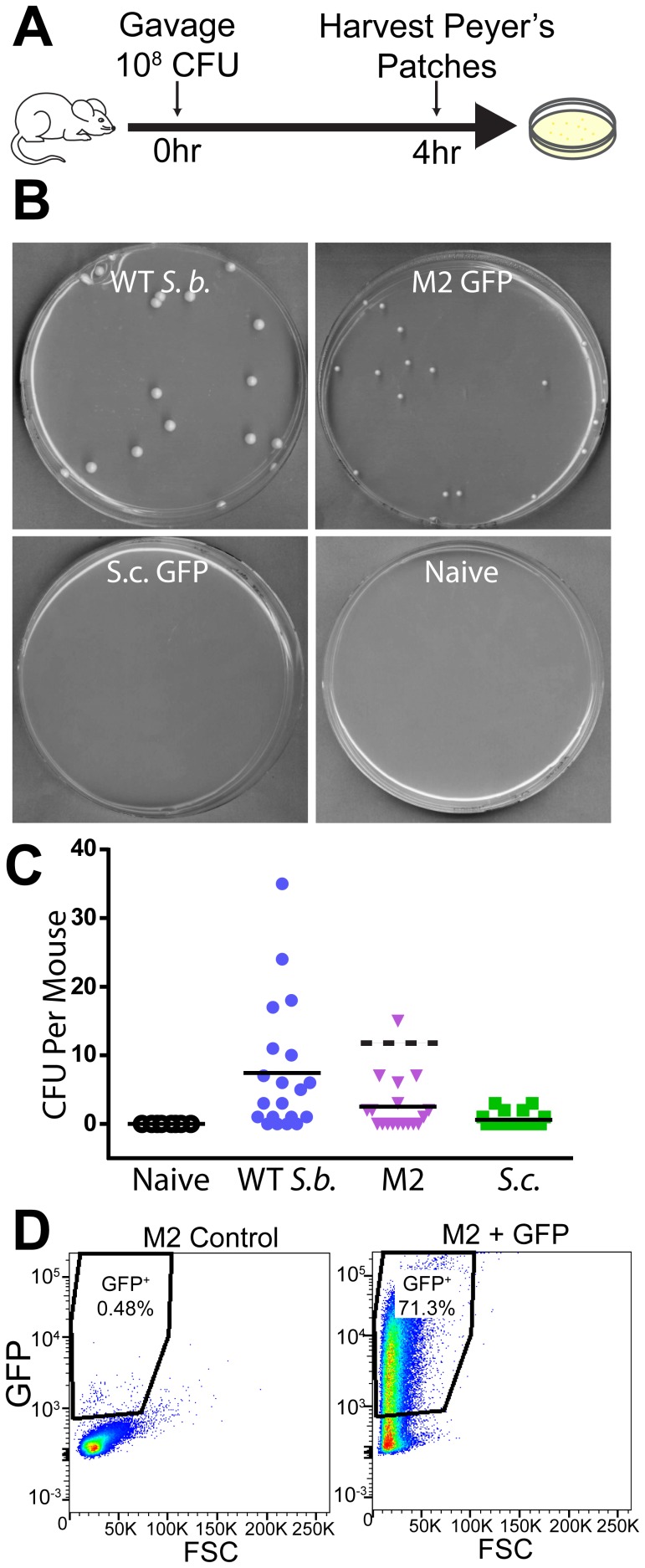
Viable Transformed *S. boulardii* Mutant 2 can be Recovered from Gastrointestinal Immune Tissue. (A) Schematic depicting oral gavage experiments. C57BL/6 mice were gavaged with 100 µL containing either water, 10^8^ CFU wild type *S. boulardii* (WT *S.b.*), 10^8^ CFU *S. boulardii* Mutant 2 (M2), or 10^8^ CFU *ura3^−^ S. cerevisiae* laboratory haploid (*S.c.*). Peyer's patches, sites of antigen sampling and immune response generation in the gastrointestinal tract (reviewed in [38]), were harvested 4 hours post gavage and plated to detect viable CFU. (B) Images of typical plates from oral gavage experiments showing recovery of viable yeast from Peyer's patches. Samples from mice gavaged with WT *S. boulardii*, *S. boulardii* Mutant 2 transformed with *URA3* plasmid, or *S. cerevisiae* laboratory haploid transformed with *URA3* plasmid were plated on media lacking uracil. Samples from naïve mice were also plated on YPD media to detect any contaminating yeast unable to grow without uracil. (C) CFU per mouse recovered from Peyer's patches of mice orally gavaged with water (Naïve), WT *S. boulardii* (WT *S.b.*), *S. boulardii* Mutant 2 (M2), or *S. cerevisiae* laboratory haploid (*S.c.*) (n = 20 mice per group). Lines show the mean CFU per mouse for each group. Two data points for *S. boulardii* Mutant 2 (87 and 110 CFU per mouse) are not depicted in order to allow better visualization of other data points. The mean without the two high points is 2.5 (shown in solid black line). The mean including the two points is 12.1 (shown in dotted line). (D) Representative flow cytometry plots of forward-scattered light (FSC) versus GFP fluorescence showing the percent of GFP positive cells among untransformed *S. boulardii* Mutant 2 (M2 control) and *S. boulardii* Mutant 2 that was transformed with a *URA3* plasmid encoding GFP (M2+GFP) and subsequently recovered from murine Peyer's patches (26 total transformed *S. boulardii* M2 CFU recovered from Peyer's patches were assessed by flow cytometry).

To test for survival of *S. boulardii* Mutant 2 in the gastrointestinal tract, C57BL/6 mice were gavaged with water (Naïve), 10^8^ CFU untransformed WT *S. boulardii*, or *S. boulardii* Mutant 2 or 10^8^ CFU *S. cerevisiae* laboratory haploid transformed with the *URA3* GFP plasmid (Fig. 8A). Peyer's patches were harvested four hours post gavage, cell strained, and plated onto selective media lacking uracil. Peyer's patches from naïve mice were also plated onto YPD to check for the presence of contaminating yeast unable to grow in the absence of uracil. After 2–5 days incubation at 30°C, plates showed no viable yeast detected in Peyer's patches of naïve mice (Fig. 8B, Naïve), few to no colonies of transformed *S. cerevisiae* (Fig. 8B, *S.c.* GFP), and many viable colonies for both transformed *S. boulardii* Mutant 2 (M2 GFP) and untransformed WT *S. boulardii* (WT *S.b.*) (Fig. 8B). These results are quantitated in Figure 8C. Viable transformed *S. boulardii* Mutant 2 furthermore showed a high percentage of GFP^+^ cells, as determined by flow cytometry (Fig. 8D). This result indicates that transformed *S. boulardii* Mutant 2 is capable of maintaining heterologous protein expression despite the lack of selective pressure and harsh growth conditions within the gastrointestinal tract.

Notably, there was a high degree of variability in number of viable CFU harvested per mouse (Fig. 8C), especially for the WT *S. boulardii* and *S. boulardii* Mutant 2 groups. As observed previously for recovery of viable WT *S. boulardii* versus *S. cerevisiae* Σ1278b and BY3 strains from murine Peyer's patches [13], this variability prevented the trend of increased viable *WT S. boulardii* and *S. boulardii* Mutant 2 versus *S. cerevisiae* from reaching statistical significance (power calculations indicate a sample size of greater than 200 mice is needed to determine a statistically significance between the Mutant 2 and *S. cerevisiae* groups, given an alpha of 0.05 and an expected significant difference of 5 CFU). Such variability could be due to numerous factors, including differences in feeding prior to oral gavage, differences in digestion and gastrointestinal motility, or margins of error in Peyer's patch dissection.

## Discussion and Conclusions

A major requirement for the development of *S. boulardii* as a viable drug delivery system is the development of strains that can maintain protein expression in the harsh digestive conditions of the gastrointestinal tract. Here, we employed UV mutagenesis and 5-FOA screening to generate three *S. boulardii* auxotrophic mutant strains that can be genetically modified and transformed without reliance on antibiotic resistance markers. Critically, *S. boulardii* Mutants 1–3 can be transformed with *URA3* plasmids and express functional recombinant protein as demonstrated by GFP fluorescence (Fig. 7A–B). *S. boulardii* Mutants 1–3 maintained expression of recombinant protein for 24 hours after removal of selective pressure (Fig. 7C), a feature that will be especially important in the context of *in vivo* drug delivery. The ability of transformed mutants to continue producing GFP even after 24 hours without selective pressure suggests their potential to express and deliver recombinant proteins during transit through the gastrointestinal tract.

*S. boulardii* has previously been used successfully to produce the mammalian anti-inflammatory cytokine interleukin 10 (IL-10) [20]. This study not only confirmed the ability of WT *S. boulardii* to successfully produce properly folded IL-10 as demonstrated by ELISA, but also showed *in vivo* functionality of this secreted cytokine. Oral gavage of transformed *S. boulardii* expressing IL-10 improved ulceration scores of mice in the dextran sodium sulfate (DSS) colitis model, although there was no difference in colonic thickening or histological score compared to controls. While this study provides proof of principle for expression of a therapeutically relevant recombinant protein by *S. boulardii*, transformation in this study was dependent on the presence of aminoglycoside resistance markers and growth in media supplemented with antibiotic. Our use of *ura3^−^* auxotrophic mutant strains of *S. boulardii* in the present study allowed for selection of transformants simply using media lacking uracil, obviating the need for antibiotic resistance markers.

A goal is to genetically engineer *S. boulardii* strains to produce uracil auxotrophy without additional mutations that could impact desirable growth properties of this clinically used probiotic strain. Although a moderate dose of UV irradiation was selected in order to mutate *URA3* while limiting the number of additional mutations, genes other than *URA3* have likely been affected in *S. boulardii* Mutants 1–3. Indeed, overall growth of the three *ura3* ^−^ *S. boulardii* mutants is reduced relative to that of WT *S. boulardii* (Fig. 5). The exact mutations responsible for this reduced growth were not determined. Generation of a *ura3* ^−^ *S. boulardii* strain in future studies using a targeted approach would allow for creation of an auxotrophic mutant while maintaining pathways responsible for the superior growth rate and immunomodulatory properties of WT *S. boulardii*.

Despite a modest impact on growth rate, the three *ura3* ^−^ *S. boulardii* mutants generated in this study show resistance *in vitro* to a wide range of pH, bile acid, and anaerobic conditions similar to that of the gastrointestinal tract (Fig. 5, 6). Growth of Mutants 1–3 in anaerobic conditions is in fact higher than for WT S. boulardii and S. cerevisiae (Fig. 6). Although growth of *S. boulardii* Mutants 1–3 in YPD is reduced relative to WT *S. boulardii* in aerobic conditions, their growth in low pH and bile acid relative to growth in normal YPD media is maintained. Resistance to low pH is a key feature of *S. boulardii*, distinguishing it from even closely related *S. cerevisiae* strains. Consistent with these findings, previous studies provide evidence that WT *S. boulardii* shows resistance to low pH [39]. Maintenance of resistance to low pH and anaerobic conditions could allow *S. boulardii* Mutants 1–3 not only to synthesize therapeutic proteins but also to serve as protective capsules for those proteins during transit through the gut. Use of these auxotrophic mutants for protein synthesis and packaging would decrease cost of oral drug development by eliminating the expensive steps of purifying and packaging proteins into capsules such as liposomes or nanoparticles.

To assess the potential for *S. boulardii* auxotrophic mutants to serve as drug delivery vehicles, we tested their ability to maintain production of recombinant protein *in vivo*. For these oral gavage experiments, we selected *S. boulardii* Mutant 2, which showed equal or superior resistance to low pH, bile acid, and anaerobic conditions relative to the other mutants (Fig. 5, 6), comparable production of GFP *in vitro* (Fig. 7A–C), and reversion to a *URA3^+^* phenotype at a rate below the limit of detection (Fig. 3D). Viable *S. boulardii* Mutant 2 was isolated within murine small intestine Peyer's patches at a frequency similar to that of WT *S. boulardii* (Fig. 8C), indicating that *in vivo* survival through the gastrointestinal tract and uptake into small intestine immune tissues is maintained for this mutant and supporting the *in vitro* data showing that this mutant is resistant to low pH and bile acid (Fig. 5). High levels of GFP expression in transformed *S. boulardii* Mutant 2 recovered from Peyer's patches furthermore indicate that this mutant is capable of delivering recombinant protein to the gastrointestinal tract and its associated immune tissues (Fig. 8D). In fact, our results in this experiment likely underestimate the number of *ura3* ^−^ *S. boulardii* Mutant 2 cells entering the Peyer's patches as our assay was capable of detecting only viable yeast; *ura3* ^−^ *S. boulardii* that entered as cellular fragments or that were phagocytosed by antigen presenting cells within Peyer's patches could not be detected. The ability of *ura3* ^−^ *S. boulardii* to be taken up into immune tissues makes it well suited for oral delivery of immunomodulatory therapeutics. For example, *ura3* ^−^ *S. boulardii* could be used to express and deliver IL-10 to immune tissues of the gastrointestinal tract and thus promote anti inflammatory immune responses in the context of inflammatory bowel disease.

In summary, the *ura3* ^−^ *S. boulardii* mutants generated in this study possess all the characteristics needed for safe and efficient use as an oral drug delivery system. These mutants can be transformed and selected using auxotrophic markers to avoid reliance on antibiotic selection. Furthermore, they can express heterologous protein to a similar level as a commonly used laboratory *S. cerevisiae* strain, as demonstrated by comparable levels of GFP fluorescence. These *S. boulardii* mutants also maintain high levels of protein expression even after prolonged incubation in nonselective media and uptake into the immune tissues of the murine gastrointestinal tract. These newly generated *S. boulardii* auxotrophic mutants are therefore good candidates for further testing as drug delivery vehicles for the treatment of gastrointestinal disorders.
